# Intensity tunable infrared broadband absorbers based on VO_2_ phase transition using planar layered thin films

**DOI:** 10.1038/srep13384

**Published:** 2015-08-21

**Authors:** Hasan Kocer, Serkan Butun, Edgar Palacios, Zizhuo Liu, Sefaattin Tongay, Deyi Fu, Kevin Wang, Junqiao Wu, Koray Aydin

**Affiliations:** 1 Department of Electrical Engineering and Computer Science, Northwestern University, Evanston, IL 60208, USA; 2 Department of Electrical Engineering, Turkish Military Academy, 06654 Ankara, Turkey; 3 School for Engineering of Matter, Transport and Energy, Arizona State University, Tempe, AZ 85287, USA; 4 Department of Materials Science and Engineering, University of California Berkeley, Berkeley, CA 94720, USA

## Abstract

Plasmonic and metamaterial based nano/micro-structured materials enable spectrally selective resonant absorption, where the resonant bandwidth and absorption intensity can be engineered by controlling the size and geometry of nanostructures. Here, we demonstrate a simple, lithography-free approach for obtaining a resonant and dynamically tunable broadband absorber based on vanadium dioxide (VO_2_) phase transition. Using planar layered thin film structures, where top layer is chosen to be an ultrathin (20 nm) VO_2_ film, we demonstrate broadband IR light absorption tuning (from ~90% to ~30% in measured absorption) over the entire mid-wavelength infrared spectrum. Our numerical and experimental results indicate that the bandwidth of the absorption bands can be controlled by changing the dielectric spacer layer thickness. Broadband tunable absorbers can find applications in absorption filters, thermal emitters, thermophotovoltaics and sensing.

Controlling, manipulating and engineering the spectral absorption properties of optical materials have been an active area of research in recent years. In particular, resonant metamaterial and plasmonic absorbers have been demonstrated to enable either narrow or broadband absorption over microwave^1^, terahertz^1^, infrared^2^,^3^,^4^,^5^, and visible^6^,^7^ bands of electromagnetic spectrum. Kirchhoff’s law of thermal radiation states that the emissivity, *E*, of a material is equal to its absorption, *A*, at thermal equilibrium in which following relation holds^8^


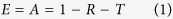


where *R* and *T* are reflectivity and transmittance, respectively. Therefore, emissive and absorptive features of a material can be controlled by the reflectivity and transmittance properties of the optical material which depend on the geometry and optical parameters of the material. Engineering the absorptivity/emissivity spectra on these windows provides spectral selectivity, which is an essential requirement for applications such as target recognition, bio-chemical sensing, camouflage, infrared (IR) signature mimicry, imaging, sensors, IR labeling and wavelength selective IR sources^9^,^10^.

Metamaterial and plasmonic based micro or nanostructured, so called sub-wavelength, absorbers are very thin compared to the operating wavelength, however they often require complex, time-consuming and expensive nanofabrication steps. Recently, use of continuous and lithography-free films has opened the door for employing ultra-thin Fabry-Perot type interference films with the advantage of requiring minimal fabrication steps^11^,^12^,^13^,^14^. With the use of these kind of ultra-thin nanocavity structures in different geometries, for instance planar layered thin film structures, several experimental and numerical studies have been rapidly emerging in the visible^12^,^15^, near-infrared^16^ and mid-infrared^9^,^11^,^17^,^18^ spectral regions. Having a resonant response covering a broad wavelength range is a desired feature for infrared broadband thermal emitters^19^, thermophotovoltaic cells^2^, as well as plasmonic scatterers^20^,^21^,^22^. In order to dynamically tune the emissivity, certain tunable materials can be employed in the design^4^. Electrical^23^, optical^24^, and thermal^25^ tunability were demonstrated as the main active tuning mechanisms. Thermally tunable materials, such as vanadium dioxide (VO_2_) and niobium dioxide (NbO_2_) are called thermochromic materials, which change their optical properties as a function of temperature^26^. VO_2_ undergoes a structural transition from an insulating phase to a metallic phase at 68 °C. This reversible phase change occurs on a sub-picosecond timescale^27^,^28^. Several experimental and theoretical studies were performed to understand the structural and optical phase change mechanisms of VO_2_^29^,^30^,^31^,^32^,^33^. If the emissivity decreases as the temperature rises, the thermochromic structure has a positive dynamic range which is desired for IR signature reduction. Opposite thermal behavior has negative dynamic range that is suitable for smart windows and space applications^26^.

Depending on the thickness of the insulator spacer layer of the structure, the incident light interferes constructively or destructively with the reflected light, therefore one can control the intensity of the reflected and transmitted electromagnetic waves. In this study, we propose and demonstrate experimentally realizable planar layered thin film broadband absorbers (BBA) that are capable of tuning the absorption in the mid-wavelength infrared (MWIR) spectrum based on VO_2_ phase transition. A thin VO_2_ film is used as the top layer of a Fabry-Perot type absorber structure. The MWIR emissivity of our design has a negative dynamic range.

## Results

### Proposed designs

**Figure 1:**
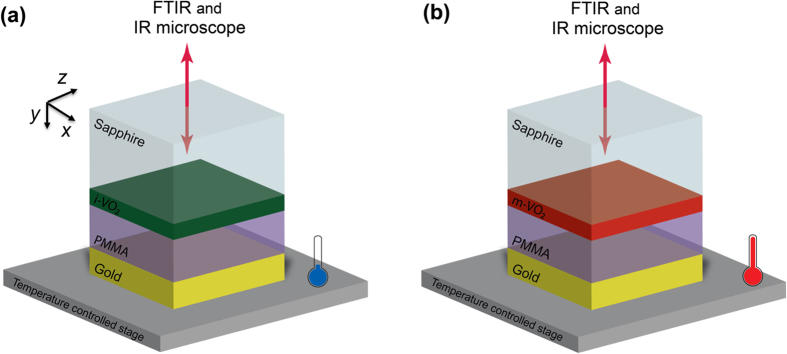
Experimental setup and BBA device designs. PMMA spacer layer (*t*_*PMMA*_ = 500 nm and 700 nm) and 60 nm gold cap layer was deposited on VO_2_ on sapphire substrate. The device is mounted upside down inside an infrared microscope and illuminated at normal incidence using a mid-IR source. (**a**) VO_2_ is set to insulator phase (i-VO_2_) by adjusting temperature of the controlled plate at 23 °C. (**b**) VO_2_ is set to metallic phase (m-VO_2_) by adjusting temperature of the controlled plate at 123 °C. (The thermometer illustration is drawn by S.B.)

Tunable BBA device designs presented here consist of three layer stack of VO_2_, dielectric and Au continuous films. Poly(methyl methacrylate) (PMMA) is used as a spacer between 20 nm VO_2_ film and a lossy 60 nm thick Au layer. Figure 1(a,b) show schematics of insulating and metallic phases of VO_2_ in connection with temperature, respectively. IR illumination is sent from the sapphire side. Au layer is placed on a temperature controlled plate, which is mounted inside an IR microscope (Bruker Hyperion 2000). Spectral reflection measurements were carried out using an IR microscope which is coupled to a Fourier transform infrared (FTIR) spectrometer (Bruker Vertex 70) equipped with liquid nitrogen cooled mercury cadmium telluride (HgCdTe) detector. Two different BBAs were designed with the PMMA thicknesses of 500 nm and 700 nm. VO_2_ film behaves like an insulator at room temperature while it is fully metallic above the transition temperature (123 °C in our study). Therefore, we will refer these two different device conditions as “i-VO_2_” and “m-VO_2_”. Temperatures of these BBAs were set to room (23 °C) and hot (123 °C) for two different cases.

### Effect of the spacer layer thickness

**Figure 2:**
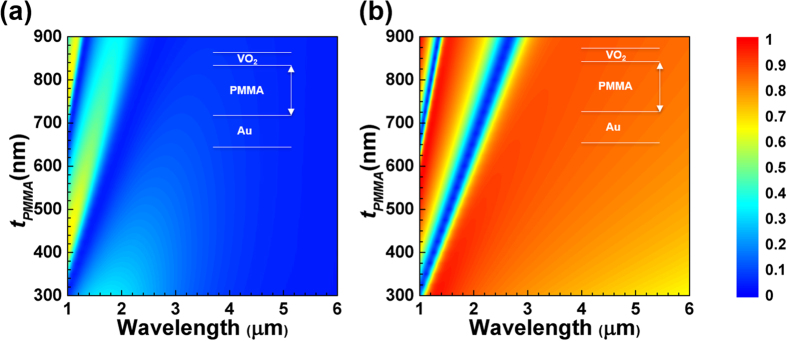
Absorption map with respect to PMMA thickness sweep for (**a**) i-VO_2_ (**b**) m-VO_2_ structure. The colorbar applies both.

Both Finite-Difference Time-Domain (FDTD) and Transfer Matrix Method (TMM) techniques are used for electromagnetic modelling. A plane wave source is assumed to be normally incident and propagate along *y*-axis through the structures. Complex refractive indices for two different phases (insulator and metal) of VO_2_ film were taken from an earlier experimental study^27^. Index of the PMMA and sapphire were set to constant values of 1.47 and 1.7, respectively. The complex refractive index of Au were taken from the Palik database^34^. The relative dielectric permittivities of the materials used in the simulations are given in Supplementary Figure S1. The effect of the thickness of the PMMA (dielectric) spacer layer were investigated using numerical simulations for i-VO_2_ and m-VO_2_ as shown in Fig. 2. Transmitted and reflected power from these absorbers were computed using transmission and reflection power monitors and the absorption was calculated by using equation (1). Simulations show that beyond the first Fabry-Perot mode, both i-VO_2_ and m-VO_2_ have a fairly constant and broadband absorption profile. However, the absorption of m-VO_2_ is substantially enhanced compared to i-VO_2_ in the MWIR spectrum. The thickness of PMMA determines the spectral position of the first order Fabry-Perot mode as expected.

### Comparisons of measurements and simulations

**Figure 3:**
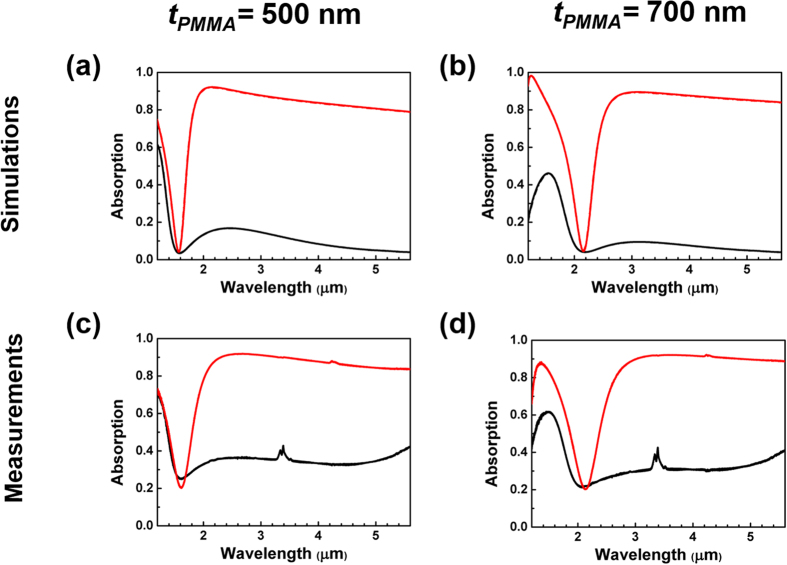
Simulated and measured absorption spectra of i-VO_2_ (black curves) and m-VO_2_ (red curves). (**a**) Simulation result for *t*_*PMMA*_ = 500 nm. (**b**) Simulation result for *t*_*PMMA*_ = 700 nm. (**c**) Measurement result for *t*_*PMMA*_ = 500 nm. (**d**) Measurement result for *t*_*PMMA*_ = 700 nm.

**Figure 4:**
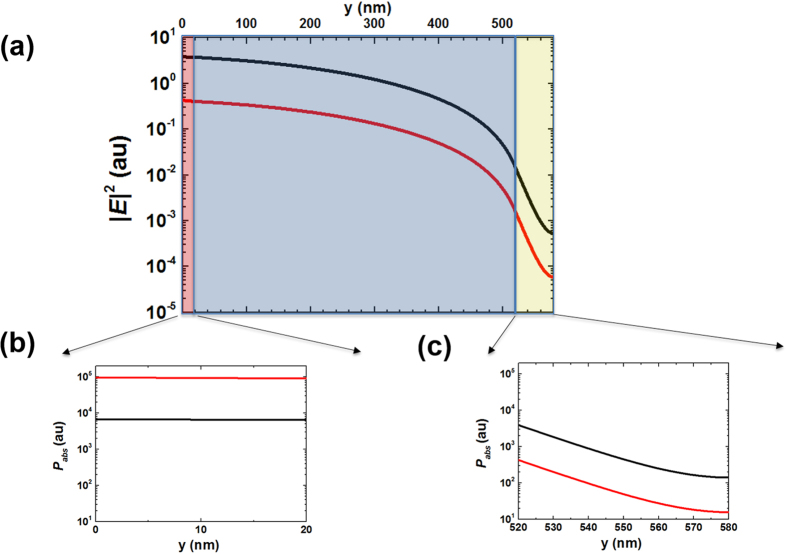
Simulated electric field intensity and absorbed power density of 500 nm thick PMMA i-VO_2_ (black curves) and m-VO_2_ (red curves) at λ = 4 μm. 20 nm-width red stripe is the VO_2_ layer, 500 nm-width blue stripe is the PMMA layer and 60 nm-width yellow stripe is the gold layer. (**a**) Electric field intensity inside the structure. (**b**) Absorbed power density along the 20 nm top VO_2_. (**c**) Absorbed power density along the 60 nm bottom Au.

In Fig. 3, we plot simulated and measured absorption spectra of i-VO_2_ (black curves) and m-VO_2_ (red curves) for the PMMA thicknesses of 500 nm (Fig. 3(a,c)) and 700 nm (Fig. 3(b,d)). For the PMMA thicknesses of 500 nm, both simulated and measured absorption spectra of i-VO_2_ and m-VO_2_ coincide around the wavelength of 1.6 μm. Beyond this wavelength, absorption intensity was shown to be dynamically changing from the near perfect absorption (~90% for the simulated and measured absorption) to the low absorption levels (~10% for the simulated and ~30% for measured absorption) in the mid-wavelength infrared spectrum based on VO_2_ phase transition. When we increase the PMMA thickness to 700 nm, the coincidence wavelength shifts to around 2.2 μm. Again, beyond this wavelength similar dynamic tunability of the absorption intensity was observed. There is a deviation in the measured absorption spectrum of the i-VO_2_ case from the simulations. This might have been resulted from two facts. Firstly, index of the experimental 60 nm thick Au structure might be somewhat different than the bulk index of the Ref. 34. Secondly, the experimental PMMA index may exhibit slightly dispersive and lossy characteristics instead of the constant one, which was not considered in our simulations. Overall, we obtained remarkably good agreement between the simulations and measurements in terms of predicting the resonant dip positions and broadband intensity tunability with respect to the changing phase of the VO_2_. When VO_2_ is insulator, IR light passes through the thin VO_2_ layer and travels into the lossless PMMA spacer layer and bounces back from the optically thick Au layer. Therefore, i-VO_2_ structure has high reflection or in other words low absorption. On the other hand, when VO_2_ becomes metallic at high temperature, increased absorption of the structure may be considered physically reasonable due to the electric field confinement and enhancement within the Fabry-Perot type nanocavity structure. Even though VO_2_ layer itself is already a temperature-tunable absorber in the MWIR range, we claim that the PMMA and Au are used to localize the electric field and amplify the VO_2_ effect as shown in Supplementary Figure S2. We also note that measured absorption spectra have two small resonant peaks at the wavelengths of 3.4 μm and 4.2 μm. The first feature around 3.4 μm is caused by the molecular vibrational absorption of PMMA itself. It is only visible in i-VO_2_ case (black curves of Fig. 3(c,d)) because in m-VO_2_ case there is an order of magnitude less electric field penetration into the PMMA layer (Fig. 4). This reduces the vibrational mode amplitude to a level which is not visible in red curves of Fig. 3(c,d). The second feature at 4.2 μm is due to the absorption of atmospheric CO_2_, an artifact related with measurement conditions. Depending on CO_2_ levels in the room at the time of sample and the reference measurements, it may or may not show up in the measured spectra.

### Absorption mechanisms of the structures

In order to better understand the absorption mechanism in VO_2_ based absorbers for two different phases, we calculated local electric fields for i-VO_2_ and m-VO_2_ absorbers at 4 μm wavelength using FDTD simulation technique, where thickness of PMMA was chosen to be 500 nm. Then, the resulting electric field intensity (|*E*|^2^) and absorbed power density (*P*_*abs*_) inside different layers were plotted in Fig. 4. *P*_*abs*_ is the divergence of the Poynting vector and for non-magnetic materials it can easily be calculated using the simple relation of


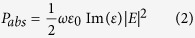


where *w* is the angular frequency, *ε*_*0*_ is the free space permittivity, Im(*ε*) is the imaginary part of the relative dielectric permittivity and |*E*| is the magnitude of the total electric field^6^,^20^. Looking at absorbed power plots (Fig. 4(b,c)), we note that dominant absorption occurs inside the 20 nm thick lossy metallic VO_2_ top layer although field intensity of the insulating one is approximately 10 times higher than the metallic case (Fig. 4(a)). This can be explained by the fact that Im(*ε*) of the metallic VO_2_ is significantly higher than its insulating phase at the wavelength of interest. We note that there is also absorption taking place inside the bottom Au-mirror layer, but this absorption is significantly lower than that of the top VO_2_ layer because of the minimal field penetration into the optically thick Au layer.

### Effect of incident angle and polarization

**Figure 5:**
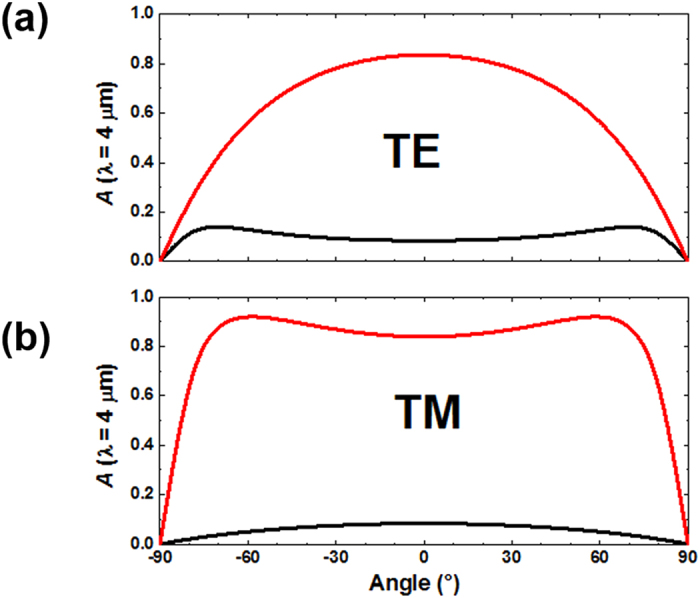
The angular dependence of absorption at *λ* = 4 μm for the structure with 500 nm thick PMMA. Black and red curves represents i-VO_2_ and m-VO_2_, respectively. (**a)** TE polarization. (**b**) TM polarization.

The angular dependence of absorption spectra for TE and TM polarizations were calculated using transfer-matrix method within the structure of 500 nm thick PMMA at 4 μm wavelength. In TE polarization, the electric field is normal to the incidence plane (*x*-*y* plane in Fig. 1), whereas it is inside the incidence plane in TM polarization. We note that TM polarization has slightly broader angular incidence and larger dynamic absorption tunability with respect to TE polarization. Nevertheless, the results in Fig. 5 show that the features of the broadband absorption and temperature tuning of the absorption are valid within a broad angular incidence of up to 60° for both polarizations.

## Discussion

We have demonstrated broadband tunable absorption (from ~90% to ~30% in measured absorption) in the mid-wavelength infrared spectrum based on VO_2_ phase transition using planar layered type thin film structures. Our design was also shown to be angular and polarization-independent IR light absorption at around 4 μm wavelength. Occurrence of the high absorption in the structure with metallic state VO_2_ were explained through the localization of electric field intensity with respect to the wavelength and the position of the device. It has been shown that increased electric field intensity within the lossy metallic VO_2_ leads to enhanced power absorption. Therefore, we can manipulate IR absorption properties by controlling the electric field localization with proper design of ultra-thin nanocavity.

## Methods

### Synthesis of VO_2_ Thin Films

The VO_2_ thin films were grown epitaxially by pulsed laser deposition on double side polished sapphire substrates at 500 °C. A hot-pressed vanadium dioxide target was used as the source material and deposition took place in 10 mTorr oxygen pressure with a growth rate of ~4 nm/min. 20 nm thick VO_2_ films were deposited with a 300 mJ laser pulse at a laser repetition rate of 5 Hz. VO_2_ films used in this study were fabricated similarly as in a report by Fu, D. *et al*. wherein a systematical study of structural, electrical transport, optical and thermoelectric properties of high-quality epitaxial VO_2_ thin films were described^35^. Sapphire substrate was chosen because of its excellent transmittance up to 6 μm.

### Fabrication of Planar Layered Thin Film Structures

Devices with two different Poly(methyl methacrylate) (PMMA) thicknesses (500 nm and 700 nm), which are schematically shown in Fig. 1 were fabricated. 500 nm and 700 nm PMMA layers were spin coated on 20 nm VO_2_. Consequently, 60 nm Au was deposited on PMMA using electron beam deposition.

### Transfer Matrix Method (TMM) Calculations

The field within each layer could be treated as superposition of forward-traveling (transmitted) and backward-traveling (reflected) wave with wave number *k* and a transfer matrix could represent the propagation through interface or within medium. According to TMM, it can be described as
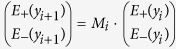
 where *M*_*i*_ can be determined by material parameters. By cascading the transfer matrix for each layer the whole system transfer matrix can be obtained, from which one could derive the transmittance (*T*) and reflectivity (*R*) of the structure^36^. Mathematical details of the incidence angle and the polarization modeling using TMM were described by Kocer *et al*.^14^.

### Finite-Difference-Time-Domain (FDTD) Simulations

Absorbed power and electric field intensity in our structures and their spectral response were calculated using commercial software from Lumerical Solutions^37^.

### Optical Measurements

Experimental reflection measurements of the structures were carried out using an infrared microscope (Bruker Hyperion 2000) and the Fourier transform infrared (FTIR) spectrometer (Bruker Vertex 70) with liquid nitrogen cooled mercury cadmium telluride and mid-IR source. Reflected light was collected using Hyperion 2000 IR microscope with a 15× magnification objective and a numerical aperture of 0.4. For the calibration of the reflection measurement, we first collected the reflection from a reference gold mirror between 1 and 6 μm. Measured reflection from the samples was then calibrated using the reflection spectra of the gold mirror. We did not measure transmission of these structures because they have an optically thick 60 nm Au bottom metal, which prevents the light transmission.

## Additional Information

**How to cite this article**: Kocer, H. *et al*. Intensity tunable infrared broadband absorbers based on VO_2_ phase transition using planar layered thin films. *Sci. Rep*. **5**, 13384; doi: 10.1038/srep13384 (2015).

## Supplementary Material

Supplementary Information
