# Supplementary material for: Intensity tunable infrared broadband absorbers based on VO2 phase transition using planar layered thin films
Source: Sci Rep. 2015 Aug 21;5:13384. doi: 10.1038/srep13384 (PMC4543955; doi:10.1038/srep13384)
Supplement: Supplementary Information [file srep13384-s1.pdf]

Supplementary Information

Intensity tunable infrared broadband absorbers based on VO_2 phase transition using planar layered thin films

Hasan Kocer[1, 2*], Serkan Butun[1], Edgar Palacios[1], Zizhuo Liu[1], Sefaattin Tongay[3], Deyi Fu[4], Kevin Wang[4], Junqiao Wu[4] & Koray Aydin[1*]

[1]Department of Electrical Engineering and Computer Science, Northwestern University, Evanston, IL 60208, USA

[2]Department of Electrical Engineering, Turkish Military Academy, 06654 Ankara, Turkey

[3]School for Engineering of Matter, Transport and Energy, Arizona State University, Tempe, AZ 85287, USA

[4]Department of Materials Science and Engineering, University of California Berkeley, Berkeley, CA 94720, USA

* E-mail: aydin@northwestern.edu; hkocer@kho.edu.tr; drhasankocer@gmail.com

1. Optical properties of materials

Real and imaginary parts of relative dielectric permittivities of VO_2 and Au, which were used in the FDTD simulations are plotted in Figure S1. Here, the data for two different phases (insulator and metal) of VO_2 film was taken from the previous experimental study (Ref. (1)) whereas Au data was taken from Palik database (Ref. (2)).

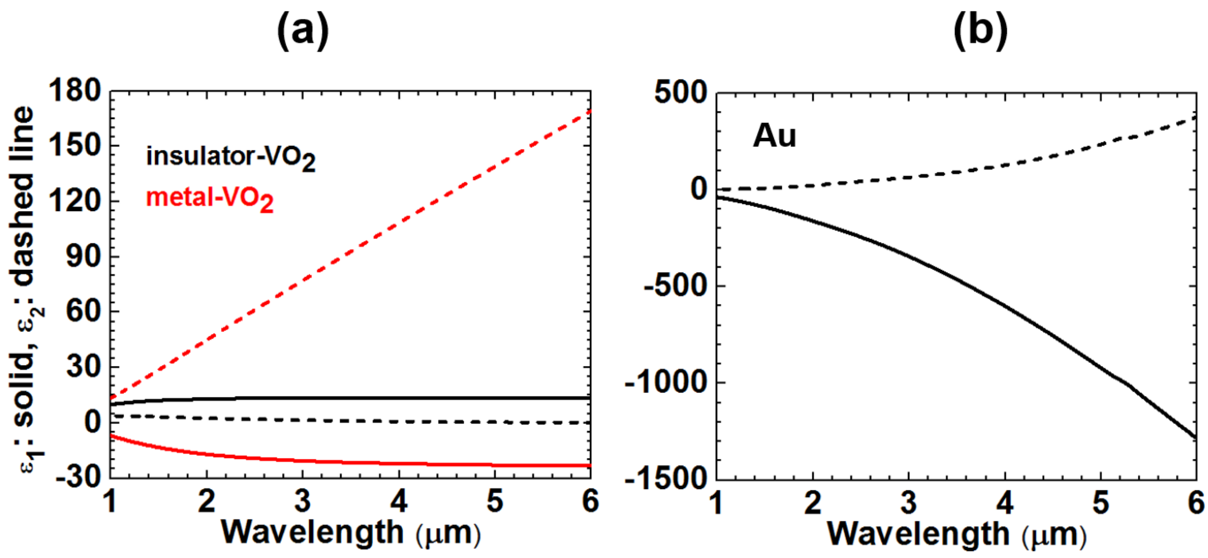

Figure S1. Wavelength dependence of real (ε_1, plotted in solid lines) and imaginary (ε_2, plotted in dashed lines) parts of relative dielectric permittivities of (a) insulating phase of VO_2 (black-colored lines) below the phase change temperature and metallic phase of VO_2 (red-colored lines) above the phase change temperature and (b) Au.

2. Comparison of VO_2 layer

Even though VO_2 layer itself is already a temperature-tunable absorber in the MWIR range, we claim that the PMMA and Au are used to localize the electric field and amplify the VO_2 effect as shown in Figure S2. Here, the absorption spectra of 20 nm VO_2 film on the sapphire substrate (Figure S2 (a)) was compared with the layered cases (Figure S2 (b) and (Figure S2 (c))).

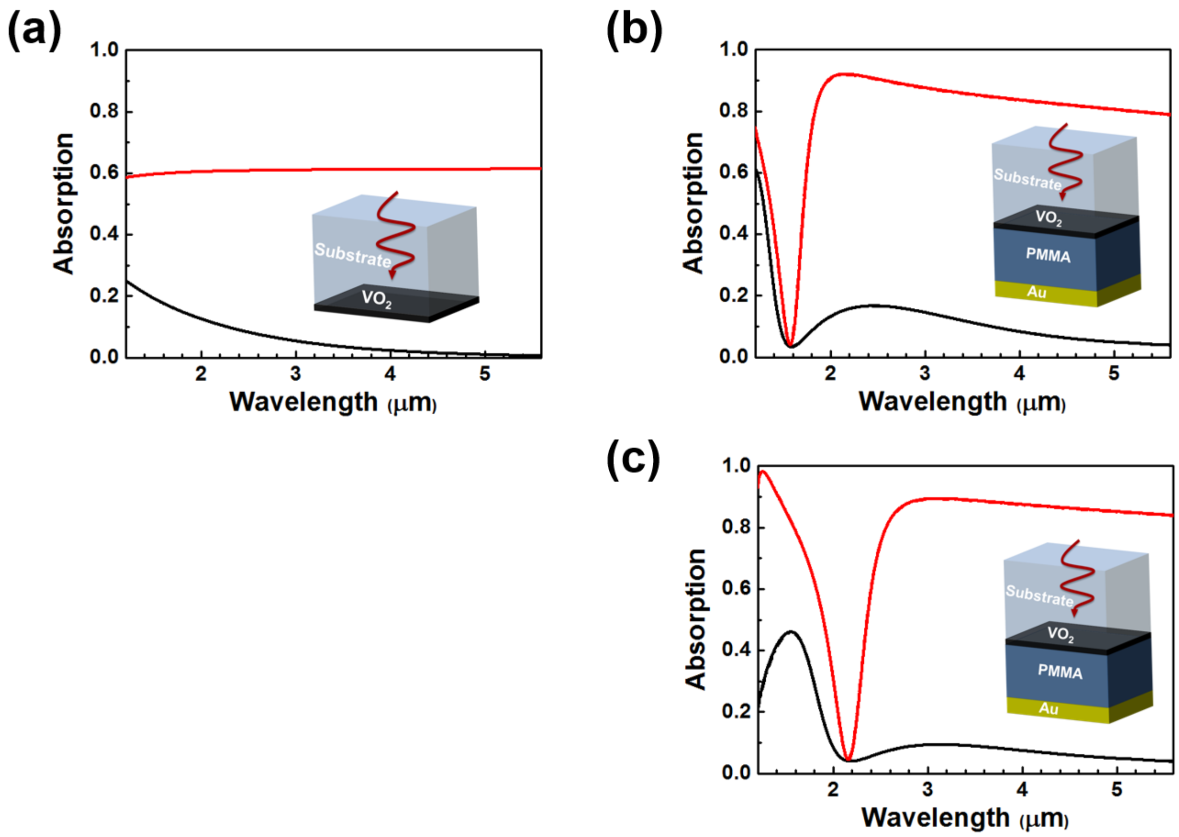

Figure S2. Simulated absorption spectra of i-VO_2 (black curves) and m-VO_2 (red curves). (a) Simulation result for 20 nm VO_2 film on the sapphire substrate. (b) Simulation result for the layered structure (20 nm VO_2 film, 500 nm PMMA and 60 nm Au) on the sapphire substrate. (c) Simulation result for the layered structure (20 nm VO_2 film, 700 nm PMMA and 60 nm Au) on the sapphire substrate.

REFERENCES

1. Dicken, M.J., et al., *Frequency tunable near-infrared metamaterials based on VO2 phase transition.* Optics Express, 2009. **17**(20): p. 18330-18339.
2. Palik, E.D., *Handbook of Optical Constants of Solids II*. 1998: Elsevier Science.
